# Dynamic Associations Between Lifestyle Factors and Positive and Negative Affect in Middle-Aged African Americans

**DOI:** 10.1093/geroni/igab046.1405

**Published:** 2021-12-17

**Authors:** Raheem Paxton, Chuong Bui, Rebecca Allen, Edward Sazonov

**Affiliations:** 1 University of Alabama, Tuscaloosa, Alabama, United States; 2 University of Alabama, University of Alabama, Alabama, United States

## Abstract

Purpose: The purpose of this study was to examine the dynamic association between lifestyle factors and both positive and negative effect in middle-aged African Americans. Methods: Study participants (N = 69, Mean age=51 years, 80% female) were recruited from two African American churches in the Deep South. Participants completed daily surveys on positive and negative effect, physical activity, sedentary behavior, diet quality, and sleep quality daily for up to 10-days. Mixed-effect models were used to examine associations between the variables of interest. Results: On days that participants were more active, they experienced higher mean positive effect (P = .015) and lower mean negative effect (P = .028) scores. Conversely, more time spent sitting in lagged models (i.e., T-1) was associated with higher mean negative effect (P = .001) and lower mean positive effect (P = .040) scores. In lagged models, better sleep quality was associated with higher positive effects (P = .007) scores but reported lower negative effects (P < .0001) scores on the same day. Lastly, on days where diet quality was higher, positive effect scores were higher (P < 0.05). Association between diet quality and positive effect was moderated by age (P = .025). Conclusion: The data suggest that same and previous day health behaviors may have a significant impact on the health and well-being of middle-aged African Americans. More research is needed to determine whether these behaviors can be targeted in real-time as a means of improving mental health outcomes in this population.

